# Oropharyngeal adverse drug reactions: knowledge, attitudes, and practice (KAP) among Italian healthcare professionals and students

**DOI:** 10.3389/fpubh.2025.1572611

**Published:** 2025-04-11

**Authors:** Gaetano La Mantia, Giulia Marcon, Martina Coppini, Fortunato Buttacavoli, Vera Panzarella, Giuseppe Colella, Annalisa Capuano, Liberata Sportiello, Gaspare Parrinello, Ilaria Morreale, Giacomo Oteri, Giuseppe Bellavia, Vittorio Fusco, Rodolfo Mauceri, Monica Bazzano, Giuseppe Seminara, Olga Di Fede, Giuseppina Campisi

**Affiliations:** ^1^Department of Precision Medicine in Medical, Surgical and Critical Care (Me.Pre.C.C.), University of Palermo, Palermo, Italy; ^2^Unit of Oral Medicine and Dentistry for Fragile Patients, Department of Rehabilitation, Fragility, and Continuity of Care, University Hospital Palermo, Palermo, Italy; ^3^Department of Biomedical and Dental Sciences and Morphofunctional Imaging, University of Messina, Messina, Italy; ^4^Department of Engineering, University of Palermo, Palermo, Italy; ^5^Multidisciplinary Department of Medical, Surgical and Dental Specialties, University of Campania “Luigi Vanvitelli”, Naples, Italy; ^6^Department of Advanced Medical and Surgical Sciences, University of Campania “Luigi Vanvitelli”, Naples, Italy; ^7^Department of Experimental Medicine, Section of Pharmacology “L. Donatelli”, University of Campania “Luigi Vanvitelli”, Napoli, Italy; ^8^Biomedical Department of Internal and Specialty Medicine, (Di.Bi.M.I.S.), A.O.U.P “Paolo Giaccone”, University of Palermo, Palermo, Italy; ^9^Clinical Pharmacology Unit, Department of Hospital General Services, University Hospital “Paolo Giaccone”, Palermo, Italy; ^10^Department of Drug, ASP Agrigento, Agrigento, Italy; ^11^Oncology Unit, Department of Medicine, Azienda Ospedaliera-Universitaria “SS Antonio E Biagio e Cesare Arrigo”, Alessandria, Italy; ^12^Translational Medicine Unit, DAIRI Department of Integration, Research and Innovation, Azienda Ospedaliera-Universitaria “SS Antonio E Biagio e Cesare Arrigo”, Alessandria, Italy; ^13^Department of Biomedicine, Neuroscience and Advanced Diagnostic, University of Palermo, Palermo, Italy

**Keywords:** pharmacovigilance, oropharyngeal adverse drug reactions (O-ADRs), knowledge, attitudes, and practices (KAP), adverse drug reaction reporting, healthcare education

## Abstract

**Introduction:**

Pharmacovigilance plays a vital role in ensuring drug safety and protecting public health. Oropharyngeal adverse drug reactions (O-ADRs) are found to be under-reported, especially by oral health professionals, limiting the identification and management of these events.

**Aims:**

This study aimed to evaluate the knowledge, attitudes, and practices (KAP) of healthcare professionals and students regarding O-ADRs and to assess their specific expertise by a self-e-learning test.

**Materials and methods:**

A cross-sectional survey was conducted using a KAP questionnaire between April 2023 and April 2024, involving 943 participants, including physicians, dentists, dental hygienists, and students. Additionally, three sets of self-e-learning tests on O-ADRs were administered. The study employed descriptive statistics, Kruskal-Wallis tests, and logistic regression to analyze factors affecting KAP and reporting behaviors.

**Results:**

Significant gaps in KAP were identified. Only 26.5% of participants demonstrated frequent best practices for reporting O-ADRs, with dentists and dental hygienists showing lower reporting rates (13.8% and 9.3%, respectively) compared to physicians (18.8%). The results of logistic regression analyses showed that practical knowledge was significantly associated with work experience (OR = 2.15, *p* = 0.026). Students exhibited the lowest levels of practical knowledge and reporting proficiency, with only 17.6% demonstrating competence. The self-e-learning test highlighted knowledge deficits: only 22.9% of participants correctly identified O-ADR associated with antiseptic mouth rinses, additional 30.2% recognized those linked to antimicrobial drugs.

**Conclusions:**

This study highlights the need for targeted educational interventions to address gaps in O-ADR knowledge and practice. Tailored training, user-friendly digital tools, and a strong pharmacovigilance culture are crucial for improving reporting rates and ensuring patient safety.

## 1 Introduction

Pharmacovigilance plays a crucial role in safeguarding public health and ensuring drug safety.

According to the World Health Organization (WHO), pharmacovigilance encompasses the science and activities involved in identifying, evaluating, understanding, and preventing adverse effects or any other issues related to drugs (1, 2).

The expansion of the global pharmaceutical market has prompted many countries to strengthen their pharmacovigilance systems to comply with international safety standards and regulatory requirements (3–5).

A significant example of this is the introduction of Good Regulatory Practices (GRP) by the WHO in 2021, aimed at harmonizing drug safety measures worldwide, highlighting the need for robust regulatory frameworks to ensure the safe use of medications (3–5).

One of the cornerstones of post-marketing surveillance is the spontaneous reporting systems for adverse drug reactions (ADRs), whose primary goal is to rapidly identify adverse reactions (6, 7).

The definition of an ADR, provided by the International Conference on Harmonization and adopted by the WHO and Food and Drugs Administration (FDA), is: “a noxious and unintended response to a drug, which occurs at doses normally used for prophylaxis, diagnosis, or therapy” (8).

Edwards et al. broaden their definition, including reactions that require treatment modification or drug discontinuation. In contrast, an adverse drug event, on the other hand, does not necessarily imply a causal relationship with the drug (9).

The pharmacovigilance systems are generally cost-effective, easy to use, and applicable to a wide range of drugs and the entire population. However, their effectiveness heavily depends on the reporting rate, which suffers from chronic under-reporting (6, 10).

Despite international efforts, significant gaps remain. For instance, in the United Kingdom, the “Yellow Card” system, operational since 1964, records that only 10–15% of serious adverse reactions are reported (11).

In 2022, the Food and Drugs Administration's Adverse Event Reporting System (FAERS) recorded over 1.25 million serious events and ~175,000 deaths related to medications (12). Approximately 38% of emergency department visits due to medication-related harm led to hospitalization, and ADRs are linked to 3 out of every 1,000 hospital deaths, with prevalence rates ranging from 5% to 20%. This also contributes to higher morbidity, mortality, and healthcare costs, highlighting the importance of identifying and preventing these events (12–15).

In Denmark, 6,109 oral adverse drug reactions (O-ADRs) were reported in 3,650 unique cases, with 70% of the cases involving women. O-ADRs accounted for ~5% of total ADRs, with an average rate of 5.8 per 100,000 people per year. The reports came from physicians (44%), dentists (19%), and citizens (10%) (16).

In Italy, the landscape is similarly challenging, with a national reporting system still under full development and significant regional disparities in reporting rates. In 1992, only 75 reports per million Italian inhabitants were sent to the WHO Collaborating Center, compared to 429 reports from Denmark and 407 from Germany (17, 18). Moreover, Italian reports were concentrated in a few regions: in 1994, 40% came from just three regions (Veneto, Friuli-Venezia Giulia, and Emilia Romagna), representing only 17% of the Italian population. This indicates that factors such as the attitudes and awareness of healthcare professionals significantly influence their active participation in pharmacovigilance systems (17).

Despite significant underreporting, particularly from oral health professionals (dentists and dental hygienists), a recent analysis conducted in the regions of Piedmont, Campania, and Sicily during the 2019–2021 period revealed a total of 3,324 reports of O-ADRs, of which 65.9% concerned conditions of the oral cavity (19).

O-ADRs under-reporting continues to be a critical issue and is often attributed to low awareness and a lack of adequate training among healthcare professionals (20).

Previous studies have shown that deficiencies in the knowledge and attitudes of healthcare professionals toward pharmacovigilance are among the main causes of O-ADR under-reporting (21). Healthcare professionals' awareness of the objectives of reporting systems is crucial for understanding the reasons behind under-reporting and for planning strategies to improve reporting rates (22).

This importance is heightened in a context where continuous changes in healthcare systems demand improvements in knowledge, attitudes, and practices (KAP) related to pharmacovigilance, particularly among healthcare professionals and students (23).

In this study, a cross-sectional survey was conducted to assess the level of KAP and specific expertise about O-ADRs among healthcare professionals and students in the fields of medicine, dentistry, and dental hygiene. This was achieved through a KAP questionnaire and a self-learning test.

## 2 Materials and methods

A cross-sectional survey was conducted with a KAP questionnaire and test self-e-learning, targeting students and health professionals, including physicians, dentists, and dental hygienists, from various regions in Italy. Data collection spanned from April 2023 to April 2024. The study protocol adhered to the ethical standards outlined in the 1964 Declaration of Helsinki and its subsequent amendments, receiving approval from the institutional review board at the University Hospital “Policlinico Paolo Giaccone” in Palermo (Ethics Committee approval no. 06/2023). Informed consent was obtained from all participants, and no financial or other incentives were provided for participation.

### 2.1 KAP questionnaire

The KAP questionnaire, administered via the Google Forms platform, was divided into four sections with the following coding structure. It was disseminated through mailing lists of medical and dental associations and presented at several national conferences to ensure wider participation. To prevent multiple responses from the same participant, the option to submit a response only once was enabled.

The structure and scoring criteria for each section are described below:

- Personal information: 6 questions related to demographic and professional details;
- Knowledge assessment: 9 questions evaluating understanding of O-ADRs (correct answer = 1, wrong answer or don't know = 0);
- Attitudes toward reporting: 7 questions examining participants' perspectives on reporting O-ADRs (disagree = 0, undecided = 1, agree = 2);
- Practical experience: 3 questions focusing on real-world experiences in reporting O-ADRs (yes = 1, no or don't know = 0).

The knowledge section is designed to evaluate participants' understanding of O-ADRs through nine statements that can be true or false. The total score is obtained by summing the scores of all items in each section, with higher scores reflecting greater levels of accurate knowledge about O-ADRs, more favorable attitudes toward the reporting of O-ADRs and greater practical experience with O-ADRs reporting. Cut-off levels for measuring KAP were identified as the third quartile of each score distribution: a cut-off level of 8 was chosen to indicate a respondent with a precise understanding of O-ADRs; a cut-off level of 14 was set to indicate a respondent with positive attitudes toward O-ADRs reporting; a cut-off level of 2 was identified to indicate more frequent best practice. Furthermore, a self-e-learning test was developed to assist participants in evaluating their clinical expertise on O-ADRs.

### 2.2 Self-e-learning test

The self-e-learning module on managing O-ADRs was designed to enable users to assess their knowledge and comprehension of O-ADRs through a series of multiple-choice questionnaires and clinical pictures. The platform consisted of three sets of tests designed to provide a customizable learning experience that is accessible to both students and dental professionals at any time and from any location. Each test was structured to be completed in ~20 min and included a total of 10 multiple-choice questions that covered various aspects of O-ADRs. To successfully pass the test, users had to achieve a minimum score of 60%, which equates to at least 6 correct answers out of 10. The question content was validated by oral medicine experts (G.C., G.L.M., and M.C.) and was systematically organized to present increasing levels of complexity, categorized into three progressive difficulty levels. This structured approach facilitates gradual skill development. After completing each test, users receive immediate and detailed feedback, including a review of correct answers and a comparison of their responses with those of other participants on the platform. This comparison feature was designed to encourage critical reflection and deeper exploration of the concepts. Furthermore, for each question, links to supplementary materials are provided, enabling users to delve deeper into the topics covered.

The tests were crafted to adapt to the user's abilities, progressively increasing the difficulty of questions based on skills demonstrated in previous levels. This adaptive system promotes constructive learning, fostering the acquisition of new knowledge while reinforcing existing understanding. The platform's flexibility allowed users to manage their learning journey, choosing the time and place that suits them best to complete the tests.

## 3 Statistical analysis

Responses were analyzed based on healthcare professional fields (physicians, dentists, dental hygienists, and students), any significant statistical differences were evaluated using the *p-*value associated to the Kruskal-Wallis' rank or Pearson's Chi-squared test, with a significance level equal to 0.05.

Descriptive statistics were used to analyze the participants' socio-demographic and characteristics and corresponding test results. In particular, self-learning scores were summarized through mean value, standard deviation (SD), median and interquartile range (IQR); multiple logistic regression model was performed to examine the relation between Practice and sociodemographic factors, residential area, Knowledge about O-ADR among students and health professionals. All results were reported in odds ratios (OR) and 95% confidence intervals (95% CI).

All statistical analysis were performed using R software (24).

## 4 Results

### 4.1 KAP questionnaire

The questionnaire was administered to 943 subjects, including 108 dental hygiene graduates, 69 physicians, 573 dentists, and 193 students from all degree courses. The survey provided detailed data on KAP, and awareness related to adverse drug reactions in the oral cavity. Results of each item are collected in Table 1, grouped by the healthcare professional field.

**Table 1:** Results of the KAP questionnaire on O-ADRs.

**Knowledge items**	**Overall *N =* 943**	**Dentists *N =* 573**	**Students *N =* 193**	**Dental hygienists *N =* 108**	**Physicians *N =* 69**	***p-*value**
**Adverse drug reactions in the oral cavity exclusively caused by the use of expired drugs or drug overdose by a patient**						0.033
0	109 (11.6%)	56 (9.8%)	33 (17.1%)	10 (9.3%)	10 (14.5%)	
1	834 (88.4%)	517 (90.2%)	160 (82.9%)	98 (90.7%)	59 (85.5%)	
**If an O-ADR occurs following the use of drugs already established as effective on the market, it is considered** **a 'medical incident'**						0.7
0	226 (24.0%)	134 (23.4%)	51 (26.4%)	23 (21.3%)	18 (26.1%)	
1	717 (76.0%)	439 (76.6%)	142 (73.6%)	85 (78.7%)	51 (73.9%)	
**According to current regulations, healthcare professionals such as doctors, dentists, dental hygienists,** **pharmacists, and nurses can independently report O-ADR information**						0.004
0	82 (8.7%)	40 (7.0%)	29 (15.0%)	6 (5.6%)	7 (10.1%)	
1	861 (91.3%)	533 (93.0%)	164 (85.0%)	102 (94.4%)	62 (89.9%)	
**Under current regulations, patients are allowed to independently report O-ADR information**						0.11
0	429 (45.5%)	277 (48.3%)	84 (43.5%)	40 (37.0%)	28 (40.6%)	
1	514 (54.5%)	296 (51.7%)	109 (56.5%)	68 (63.0%)	41 (59.4%)	
**An O-ADR can only be reported when it is confirmed that the adverse reaction is caused by drug intake**						0.001
0	451 (47.8%)	249 (43.5%)	114 (59.1%)	57 (52.8%)	31 (44.9%)	
1	492 (52.2%)	324 (56.5%)	79 (40.9%)	51 (47.2%)	38 (55.1%)	
**As a healthcare professional or student, I have/will have sufficient knowledge to identify a suspected O-ADR**						<0.001
0	362 (38.4%)	201 (35.1%)	64 (33.2%)	68 (63.0%)	29 (42.0%)	
1	581 (61.6%)	372 (64.9%)	129 (66.8%)	40 (37.0%)	40 (58.0%)	
**Oral leukoplakia is an O-ADR**						<0.001
0	152 (16.1%)	69 (12.0%)	53 (27.5%)	14 (13.0%)	16 (23.2%)	
1	791 (83.9%)	504 (88.0%)	140 (72.5%)	94 (87.0%)	53 (76.8%)	
**Gingival enlargement can be an O-ADR**						0.015
0	56 (5.9%)	26 (4.5%)	14 (7.3%)	6 (5.6%)	10 (14.5%)	
1	887 (94.1%)	547 (95.5%)	179 (92.7%)	102 (94.4%)	59 (85.5%)	
**Osteonecrosis of the jaw can be an O-ADR**						0.7
0	145 (15.4%)	84 (14.7%)	30 (15.5%)	17 (15.7%)	14 (20.3%)	
1	798 (84.6%)	489 (85.3%)	163 (84.5%)	91 (84.3%)	55 (79.7%)	
Attitude Items	Overall *N =* 943	Dentists *N =* 573	Students *N =* 193	Dental hygienists *N =* 108	Physicians *N =* 69	*p-*value
**O-ADR reports sent to AIFA can improve drug safety**						0.2
0	4 (0.4%)	2 (0.3%)	2 (1.0%)	0 (0.0%)	0 (0.0%)	
1	22 (2.3%)	13 (2.3%)	6 (3.1%)	0 (0.0%)	3 (4.3%)	
2	917 (97.2%)	558 (97.4%)	185 (95.9%)	108 (100.0%)	66 (95.7%)	
**Gathering information on O-ADR through reports sent to AIFA is advantageous for patients and benefits everyone**						0.14
0	2 (0.2%)	0 (0.0%)	2 (1.0%)	0 (0.0%)	0 (0.0%)	
1	19 (2.0%)	10 (1.7%)	7 (3.6%)	1 (0.9%)	1 (1.4%)	
2	922 (97.8%)	563 (98.3%)	184 (95.3%)	107 (99.1%)	68 (98.6%)	
**It is important for healthcare professionals to provide patients with detailed information on O-ADR**						0.055
0	11 (1.2%)	6 (1.0%)	2 (1.0%)	0 (0.0%)	3 (4.3%)	
1	36 (3.8%)	20 (3.5%)	12 (6.2%)	1 (0.9%)	3 (4.3%)	
2	896 (95.0%)	547 (95.5%)	179 (92.7%)	107 (99.1%)	63 (91.3%)	
**It is essential to promote the importance of O-ADR reporting to the general public**						–
0	19 (2.0%)	9 (1.6%)	6 (3.1%)	1 (0.9%)	3 (4.3%)	
1	72 (7.6%)	48 (8.4%)	10 (5.2%)	6 (5.6%)	8 (11.6%)	
2	852 (90.3%)	516 (90.1%)	177 (91.7%)	101 (93.5%)	58 (84.1%)	
**It is important to regularly read news and reports published on the AIFA website**						0.3
0	7 (0.7%)	3 (0.5%)	3 (1.6%)	0 (0.0%)	1 (1.4%)	
1	29 (3.1%)	22 (3.8%)	5 (2.6%)	1 (0.9%)	1 (1.4%)	
2	907 (96.2%)	548 (95.6%)	185 (95.9%)	107 (99.1%)	67 (97.1%)	
**O-ADR reporting should be considered a personal professional responsibility**						–
0	20 (2.1%)	11 (1.9%)	6 (3.1%)	1 (0.9%)	2 (2.9%)	
1	98 (10.4%)	60 (10.5%)	16 (8.3%)	16 (14.8%)	6 (8.7%)	
2	825 (87.5%)	502 (87.6%)	171 (88.6%)	91 (84.3%)	61 (88.4%)	
**It would be very useful if AIFA, in addition to its website, provided an easy-to-use app for reporting ADRs**						0.05
0	8 (0.8%)	4 (0.7%)	4 (2.1%)	0 (0.0%)	0 (0.0%)	
1	38 (4.0%)	18 (3.1%)	14 (7.3%)	2 (1.9%)	4 (5.8%)	
2	897 (95.1%)	551 (96.2%)	175 (90.7%)	106 (98.1%)	65 (94.2%)	
**Practice items**	**Overall** ***N** =* **943**	**Dentists** ***N** =* **573**	**Students** ***N** =* **193**	**Dental hygienists** ***N** =* **108**	**Physicians** ***N** =* **69**	***p-*** **value**
**Have you ever reported a suspected adverse drug reaction?**						0.005
0	830 (88.0%)	494 (86.2%)	182 (94.3%)	98 (90.7%)	56 (81.2%)	
1	113 (12.0%)	79 (13.8%)	11 (5.7%)	10 (9.3%)	13 (18.8%)	
**Do you know how to report a suspected ADR?**						<0.001
0	677 (71.8%)	387 (67.5%)	159 (82.4%)	88 (81.5%)	43 (62.3%)	
1	266 (28.2%)	186 (32.5%)	34 (17.6%)	20 (18.5%)	26 (37.7%)	
**Are you aware that, regardless of your personal expertise, you can report any suspected ADR,** **not only those affecting the oral cavity?**						0.006
0	390 (41.4%)	233 (40.7%)	94 (48.7%)	46 (42.6%)	17 (24.6%)	
1	553 (58.6%)	340 (59.3%)	99 (51.3%)	62 (57.4%)	52 (75.4%)	

Each cell contained n (%), *p*-value corresponded to Pearson's Chi-squared test or Fisher's exact test.

– *P*-value omitted, not available.

The geographical distribution of participants indicated a higher concentration of physicians and dental hygienists in the northwest of Italy, whereas dentists and students were primarily located in the south. Work experience correlated with participant's age: students had no practical experience, while physicians had the most seniority, with over 30% reporting more than 20 years of professional experience.

The analysis of knowledge regarding O-ADRs revealed significant differences among the various education levels (*p-*value = 0.027). Dentists were the most informed about the current regulations on reporting adverse drug reactions: 37.7% with more accurate knowledge, compared to 33.3% and 31.9% of dental hygienists and physicians, respectively. However, there were common gaps in awareness, particularly regarding the fact that an ADR can be reported even without absolute certainty that the drug caused the reaction. Notably, dental hygienists reported lower confidence in identifying suspected O-ADRs, while students scored the lowest in recognizing specific conditions, mistakenly identifying oral leukoplakia as an oral adverse reaction.

Regarding the attitude toward reporting, dental hygienists demonstrated greater awareness, with 81.5% stating they were aware of the possibility of reporting any suspected O-ADR. However, both dentists and dental hygienists were the least likely to report, with reporting rates of 13.8% and 9.3%, respectively, compared to 18.8% among physicians (*p-*value = 0.005), which remains low overall. Despite a higher theoretical awareness among physicians, practical knowledge of how to report an O-ADR was generally inadequate across all groups.

**Figure 1:**
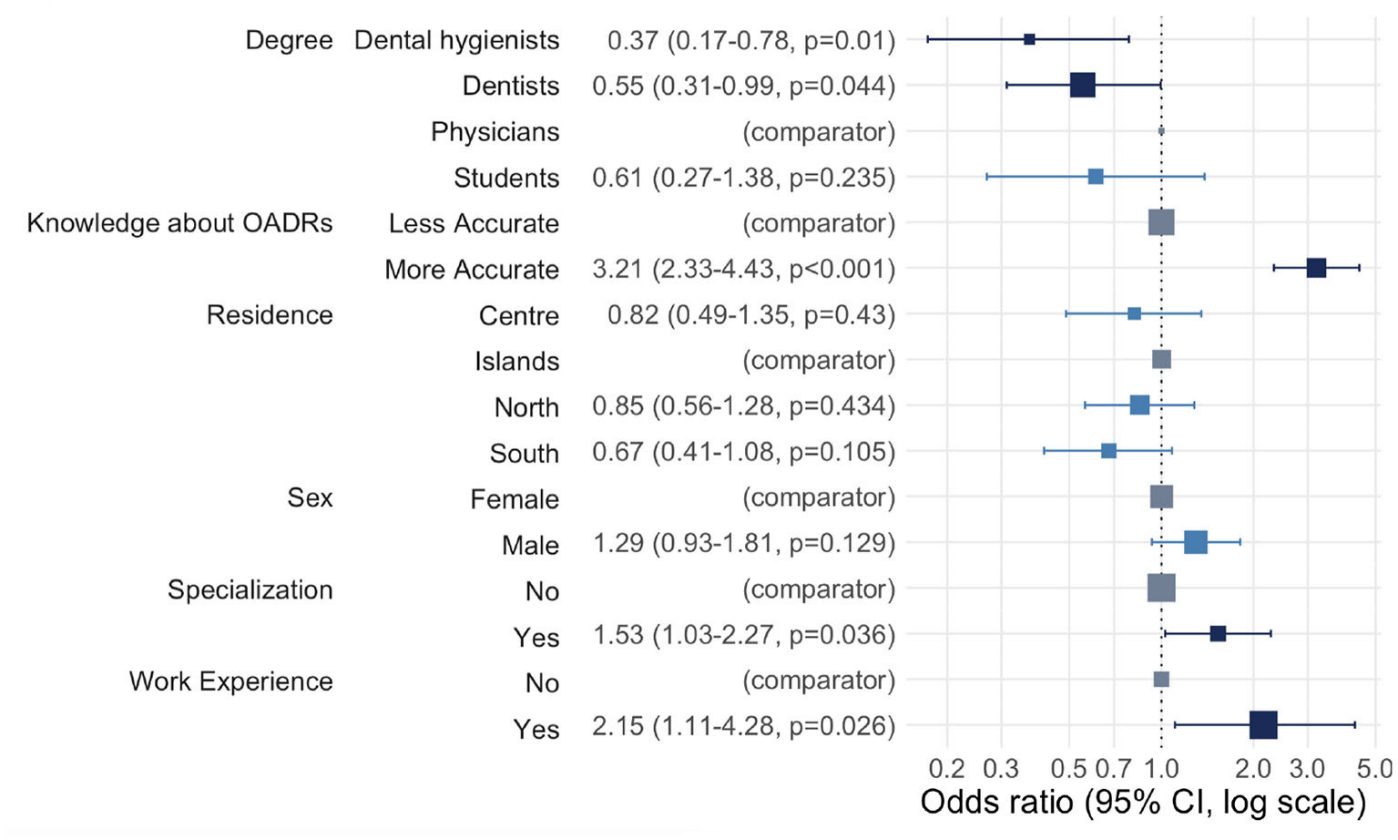
Multiple logistic regression results: practice toward reporting O-ADR.

Figure 1 shows multiple logistic regression results for practice toward reporting a suspected adverse drug reaction (O-ADR): all odds ratios (OR) >1 established more frequent best practices. Both oral healthcare professionals (dentists and dental hygienists) and students demonstrated less frequent best practices than physicians. However, the relationship appeared statistically significant only for dental hygienists and dentists (OR = 0.37, *p-*value = 0.01 for dental hygienists and OR = 0.55, *p-*value = 0.04 for dentists). As expected, the odds of having more frequent best practices were 3.21 higher among individuals who had more accurate knowledge than among individuals who had less accurate knowledge (*p-*value < 0.001). No significant association was shown for the socio-demographic characteristics, residence and gender. Individuals with specialization were 1.53 times more likely to report suspected O-ADR than individuals without it (*p-*value = 0.036). The odds of more frequent best practices for individuals with work experience were 2.15 times greater than for individuals without any work experience (*p-*value = 0.026).

### 4.2 Self-e-learning test

With respect to the self-learning tests access was granted only after completing the KAP questionnaire via a dedicated link. Out of 943 participants, 328 completed Test 1, 139 took Test 2, and 97 participated in Test 3 (see Table 2). Detailed results are collected in the supplementary section.

**Table 2:** Self-e-learning tests' results.

**Test**	***N***	**Pass (%)**	**Mean (SD)**	**Median [IQR]**
1	328	192 (59%)	74 (14)	70 [60–90]
2	139	79 (57%)	79 (16)	70 [60–100]
3	97	56 (58%)	77 (15)	80 [60–90]

Analyzing the score distribution reveals significant differences between the various tests. Percentages responses' of each item are displayed.

**Figure 2:**
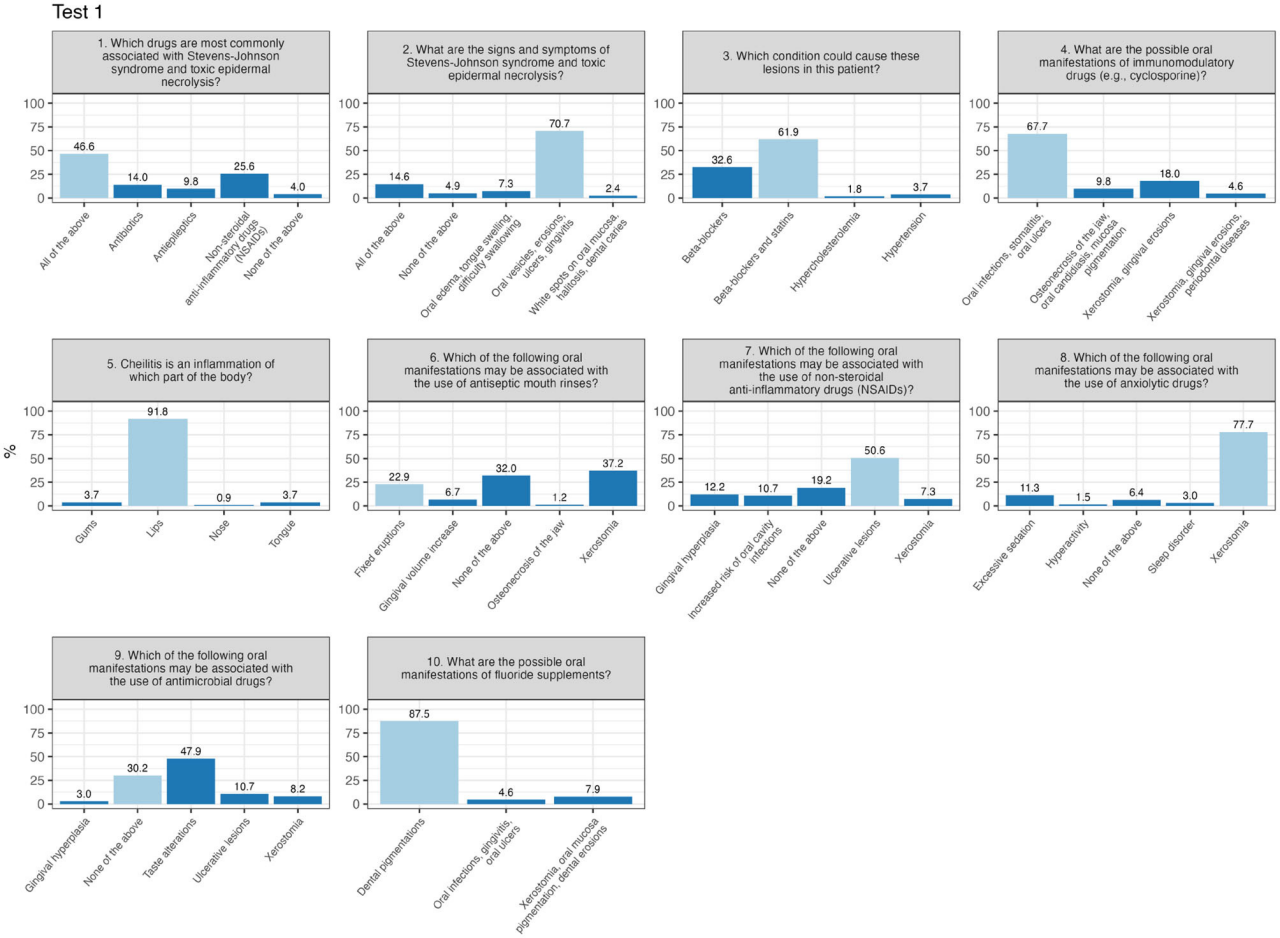
Percentage of responses for each item of test 1 (correct response in light-blue).

Test 1 shows a higher concentration of scores near the passing threshold, suggesting that many participants barely met the minimum required to pass. In Test 1 (Figure 2), certain questions raised issues due to a high percentage of incorrect answers. Question number 6 (“Which of the following oral manifestations may be associated with the use of antiseptic mouth rinses?”) was answered correctly by only 22.9%, with most choosing incorrect responses. Similarly, question number 9 (“Which of the following oral manifestations may be associated with the use of antimicrobial drugs?”) saw only 30.2% answering correctly, while 47.9% selected a common incorrect option.

**Figure 3:**
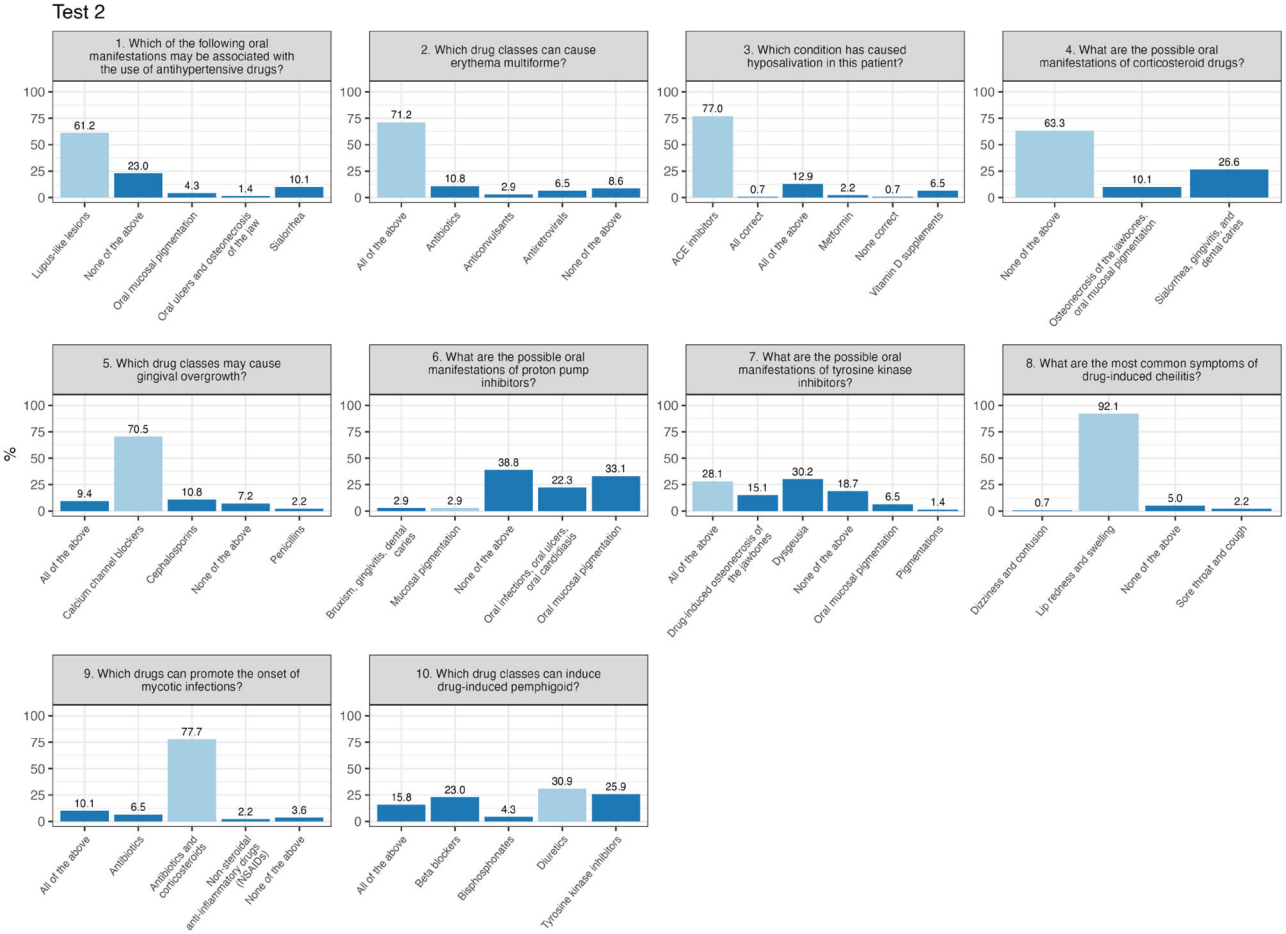
Percentage of responses for each item of test 2 (correct response in light-blue).

In contrast, Test 2 presents a bimodal distribution, with peaks at both lower and higher scores, indicating a greater variability in participants performance. In Test 2 (Figure 3), critical questions included question 6 (“What are the possible oral manifestations of proton pump inhibitors?”), with just 2.9% answering correctly, and question number 10 (“Which drug classes can induce drug-induced pemphigus?”), where only 30.9% provided the correct response.

**Figure 4:**
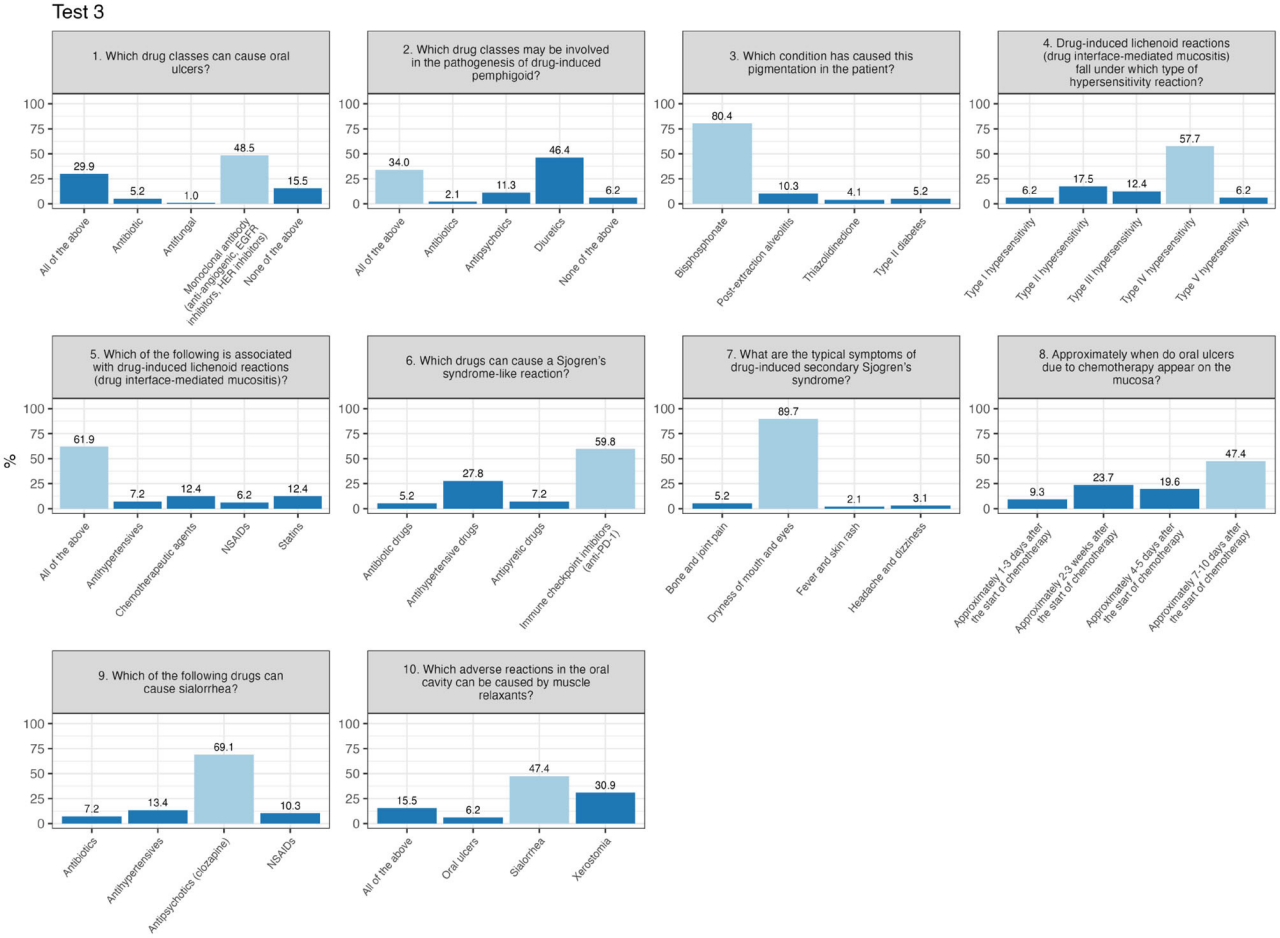
Percentage of responses for each item of test 3 (correct response in light-blue).

Finally, Test 3 displays an almost even distribution of scores ranging from 60 to 100, reflecting a wide range of results and more diverse levels of preparation among participants. There are no distinct peaks or significant concentrations at either low or high values. In other words, the scores are distributed evenly across the entire spectrum, ranging from the minimum passing mark to the maximum score. In Test 3 (Figure 4), the most challenging item was question number 2 (“Which drug classes may be involved in the pathogenesis of drug-induced pemphigus?”), with 34% answering correctly, while 46.4% selected the same incorrect alternative.

## 5 Discussion

To the best of our knowledge, this research represents the first national survey conducted in Italy aimed at exploring the levels of KAP, as well as the specific expertise of healthcare professionals and students regarding O-ADRs.

Regarding knowledge of O-ADRs, data analysis revealed significant variations in the understanding of O-ADRs across different professional groups. Physicians and dentists demonstrated a better grasp of pharmacovigilance regulations compared to dental hygienists and students. Gizem Colakoglu et al. reported similar findings in their study of dentists in Turkey, where a substantial number could correctly define concepts like pharmacovigilance (64.7%) and ADR (74.9%) (25). Conversely, in the study by Khan et al., ~50% of healthcare professionals in Pakistan showed limited knowledge, underscoring the need for more structured training (21).

In our study, dental hygienists and students struggled to recognize O-ADRs and correctly apply reporting procedures. Ohaju-Obodo et al., in a survey of 971 medical residents in Nigeria, found that 78.1% had insufficient knowledge of pharmacovigilance, reflecting comparable issues (26).

To address these gaps, academic curricula must be revised to include pharmacovigilance modules early in training. These modules should incorporate clinical simulations and hands-on exercises to equip students with practical skills for managing and reporting ADRs. Integrating an interdisciplinary approach that blends theoretical and practical learning is crucial to adequately prepare future healthcare professionals (27, 28).

In terms of attitude and practice in O-ADR reporting, our study revealed a noticeable gap between knowledge and its practical application in reporting ADRs.

While physicians, dentists, and dental hygienists were generally aware of their responsibility to independently report adverse reactions, only 32.5% of dentists and 18.5% of dental hygienists knew the correct procedures for reporting an ADR, compared to 37.7% of physicians. Furthermore, only 13.8% of dentists and 9.3% of hygienists had ever reported an ADR to the Italian Medicines Agency (AIFA), compared to 18.8% of physicians.

These results align with Khan et al. (2023), where only 13% of healthcare professionals had reported an ADR despite being aware of the reporting procedures (21). In a study by Gizem Colakoglu et al., only 2.8% of dentists reported an ADR during their career, despite 52.5% having encountered oral ADRs, with more experienced specialists being more likely to identify them (25).

To overcome these challenges, healthcare institutions should implement targeted strategies, such as intuitive digital reporting platforms that simplify the process and reduce administrative burdens. Additionally, raising awareness about the significance of ADR reporting for drug safety is essential. Establishing a reporting culture that acknowledges, and rewards healthcare professionals' contributions could encourage more proactive behavior (29–31).

Self-assessment tests revealed significant shortcomings not only in participants' practical knowledge of recognizing O-ADRs and their causes but also in the management of these reactions. Although many showed a theoretical understanding of ADRs, this knowledge did not translate into sufficient competence in clinical practice. The percentage of correct answers concerning oral manifestations induced by common drugs, such as antiseptic mouthwashes and proton pump inhibitors, was low. This indicates that theoretical awareness of ADRs is not backed by the operational understanding needed for effective clinical application.

These findings emphasize the importance of ongoing, targeted practical oral medicine training to develop advanced competencies in managing O-ADRs. Moreover, awareness alone, without accompanying practical skills, rarely translates into effective clinical practice. Even among experienced professionals, difficulties were noted in recognizing less common conditions, such as drug-induced pemphigus, highlighting that theoretical knowledge does not always equate to practical mastery.

To address the issue of under-reporting and improve drug safety, it is essential to enhance education in healthcare degree programs and promote continuous professional development for healthcare providers, reinforcing their awareness of pharmacovigilance procedures (20, 32, 33).

Worthy of note, all healthcare professionals are obligated to actively participate in pharmacovigilance activities to ensure patient safety (34, 35). In our study, over 85% of participants recognized that reporting ADRs is a fundamental professional responsibility. However, a concerning finding emerged: only a minority of dentists could effectively recognize and report an oral ADR. This highlights a critical gap between awareness and practical application, suggesting the need for more effective and targeted educational strategies (36).

Pharmacovigilance activities should extend beyond theory to become a rigorous clinical practice, supported by continuous education and accessible, user-friendly reporting tools (29).

Bridging this gap is essential, as timely reporting of O-ADRs can prevent further complications and enhance drug safety. Furthermore, fostering a culture where pharmacovigilance is viewed not just as an obligation but as an integral part of professional practice can promote positive change. Dentists should be supported through awareness campaigns, practical workshops, and access to up-to-date resources to effectively recognize and manage O-ADRs in accordance with best practices. (37–39).

## 6 Factors influencing ADR reporting

Underreporting of ADRs is a global issue, and the reasons why dentists fail to report are complex and multifaceted. A key barrier is the lack of comprehensive knowledge about drugs and ADRs, making it challenging for professionals to identify and manage adverse reactions correctly. Additionally, the reluctance to discuss ADRs with colleagues is a limiting factor (40).

Many healthcare professionals fear judgment or raising unfounded concerns, leading to a preference for avoiding the topic. This reluctance undermines the creation of a collaborative environment necessary for effective pharmacovigilance. To overcome these barriers, improving training is crucial. Courses should be designed to provide a deep understanding not only of the theory behind ADRs but also of how to apply it in clinical practice. Furthermore, it is vital to create an environment where healthcare professionals are encouraged to share their experiences and discuss ADRs without fear. Training programs and national and international workshops should also emphasize the importance of pharmacovigilance as an integral part of patient safety, making clear that ADR reporting can positively impact public health (41, 42).

## 7 Limitations

This study presents some limitations that warrant consideration. Firstly, the cross-sectional design restricts the ability to assess temporal changes or trends, thereby limiting longitudinal insights into knowledge, attitudes, and practices. Secondly, the reliance on self-reported data introduces the potential for response bias, as participants may have overestimated their knowledge or reported attitudes and practices more favorably than reality. Additionally, the anonymous nature of the self-e-learning test prevented the ability to trace the demographic details of participants who completed it.

Moreover, the limitations of anonymous self-assessment through e-learning modules should be further explored, particularly concerning potential biases and limited reliability. Lastly, the sample, predominantly composed of professionals and students from southern Italy, may not fully represent other regions or international contexts, thus affecting the generalizability of the findings.

## 8 Conclusion

The study highlights significant differences between the various groups in knowledge, attitudes, and practices related to pharmacovigilance, particularly in reporting O-ADRs. Healthcare professionals demonstrated a general awareness of the importance of pharmacovigilance. Dentists showed less familiarity with reporting procedures and reported ADRs less frequently compared to physicians. Similarly, students and dental hygienists were found to require deeper knowledge and more comprehensive training in this area.

Finally, promoting a shared and inclusive pharmacovigilance culture among healthcare professionals is vital to improving patient safety and achieving greater consistency in reporting practices. Future research should focus on assessing the impact of specific educational programs designed to address these gaps and align competencies across various professional categories.

## Data Availability

The original contributions presented in the study are included in the article/supplementary material, further inquiries can be directed to the corresponding author.
